# Simple life, simple minds? How habitat simplification in aquatic ecosystems shape fish cognition

**DOI:** 10.1007/s10071-025-02042-0

**Published:** 2026-02-04

**Authors:** Annabell Klinke, Culum Brown

**Affiliations:** Department of Biological Sciences, Macquarie University, Sydney, 2109 Australia

**Keywords:** Habitat complexity, Fish cognition, Personality traits, Brain morphology, Habitat simplification

## Abstract

This narrative review synthesises current knowledge on the effects of habitat complexity, defined as the variation in physical structures within ecosystems, on fish cognition, brain morphology, and personality. Globally, habitat complexity is declining through a process known as habitat simplification, with largely unknown effects on animal cognition. Owing to their remarkable diversity and capacity for neural plasticity, fish provide an ideal model for investigating these cognitive consequences. We first examine how habitat complexity shapes brain structure both between and within species. The results highlight that species from more complex habitats often evolve larger brains and specific brain regions, like the telencephalon and cerebellum, which are crucial for advanced cognitive and motor functions. Conversely, a lack of structural complexity, such as experienced in hatchery environments, can lead to smaller brains in fishes, though this effect can be mitigated by physical environmental enrichment. The paper then explores the impact of habitat complexity on four key areas of fish cognition, i.e., general cognition including simple learning and executive functions, spatial cognition, social cognition, and numeracy. We then propose an intricate three-way interaction between habitat complexity, personality, and cognition, whereby changes in habitat complexity can indirectly alter cognition via changes in personality. The review concludes by identifying areas for future research and warns that the continued simplification of aquatic habitats may pose a significant threat to the cognitive abilities and adaptive capacity of fish in an increasingly altered world.

**Supplementary Information:**

The online version contains supplementary material available at 10.1007/s10071-025-02042-0.

## Introduction

Habitat simplification is the process of declining structural complexity in ecosystems following anthropogenic impacts (St. Pierre and Kovalenko 2014; Pérez-Toledo et al. 2024). The loss of habitat complexity, here defined as the variation in physical structures within ecosystems, has major consequences for entire ecosystems. For instance, declining habitat complexity is often reflected in the biotic homogenization of ecological communities as well as genetic and functional similarity within communities over time (McKinney and Lockwood 1999; Olden and Rooney 2006). Moreover, habitat simplification often coincides with the invasion of terrestrial and aquatic ecosystems by habitat generalists and non-native species (Marvier et al. 2004; Olden et al. 2011; Alexander et al. 2015; Tramonte et al. 2019). In terrestrial ecosystems, the loss of habitat complexity has led to increased similarity in ecological communities in terms of taxonomic, functional, and phylogenetic diversity across various groups, including plants (Keith et al. 2009), insects (Wilson et al. 2020; Pérez-Toledo et al. 2024), mammals (Isaac et al. 2014; Almeida-Maués et al. 2022; de Abreu Pestana et al. 2023), reptiles (Palmeirim et al. 2017; Nordberg and Schwarzkopf 2019; Delaney et al. 2021), amphibians (Nowakowski et al. 2018; Delaney et al. 2021; Dehling and Dehling 2023), and birds (Shimelis et al. 2013; Curtis et al. 2022). Similar trends have been observed in freshwater and marine ecosystems, where decreasing habitat complexity induced functional homogenization in invertebrate and vertebrate communities along with a decline in taxonomic diversity (Newman et al. 2015; Petsch 2016; González-Trujillo and Alonso-Moreno 2020; Kochan et al. 2023; Nalley et al. 2024).

The drivers of declining habitat complexity in aquatic ecosystems are manifold and can be separated into direct and indirect anthropogenic activities. Direct anthropogenic activities leading to a loss of habitat complexity include damming (Chen et al. 2023), river channelization (Dutta et al. 2018), the construction of artificial structures such as concrete seawalls and piers (Able and Duffy-Anderson 2005; Airoldi et al. 2005; Baxter et al. 2023), unsustainable fishing practices like trawling and dredging, as well as aquaculture (Turner et al. 1999; Chong and Sasekumar 2002), and deep sea mining (Niner et al. 2018). Indirect anthropogenic activities contributing to habitat simplification include, among others, climate change, sedimentation and eutrophication (Walters et al. 2003; Bozec et al. 2015; Pazzaglia et al. 2020). As a result, these anthropogenic activities not only alter habitat complexity, but also have cascading effects on community assemblages and the ecology, physiology, behaviour, and possibly cognition of many aquatic organisms.

Fish are an ideal model system for understanding the cascading effects of declining habitat complexity given their diversity and the wide range of habitats they occupy. Habitat complexity plays a key role in fish ecology, supporting higher biodiversity and enhancing community persistence (Luckhurst and Luckhurst 1978; Carr et al. 2002; Lingo and Szedlmayer 2006; Smokorowski and Pratt 2007; Kovalenko et al. 2012; do Nascimento et al. 2022). In addition, habitat complexity affects fish at every level of organization, from individuals and social groups to shoals, populations, and entire communities (Soukup et al. 2022). On the level of communities and populations, habitat complexity significantly affects trophic dynamics, impacting predation rates, prey selection, and competition (Ryer 1988; Beukers and Jones 1998; Almany 2004; Soukup et al. 2022). Additionally, shifts in habitat complexity alter fish recruitment patterns and post-recruitment survival (Connell and Jones 1991; Feary et al. 2007; Smokorowski and Pratt 2007). Habitat complexity also influences an individual’s stress response, energy expenditure, and thus overall fitness (Magel et al. 2017; Castejón-Silvo et al. 2021; Fakan et al. 2023; Vicente et al. 2024). Furthermore, habitat changes drive modifications in key behaviours, including antipredator responses, agonistic and social interactions, shoaling, and mating behaviour (Orpwood et al. 2008; Myhre et al. 2013; Silva-Pinto et al. 2020; Gunn et al. 2022; Keith et al. 2023; Fakan et al. 2023; Talagala et al. 2024).

Crucially, habitat complexity is positively correlated with enhanced cognitive abilities in fish (Shumway 2008; Brown 2012; White and Brown 2015a; Axelrod et al. 2021; Boesch 2021). Hence, habitat simplification may have unforeseen effects on fish cognition, particularly given the well-documented neuroplasticity of teleosts (Ebbesson and Braithwaite 2012; Fong et al. 2019). Behavioural plasticity, influenced by cognitive capacity is the first mode of response to environmental change. Yet as habitat complexity declines, cognitive capacity is potentially threatened, reducing adaptive capacity when most needed. While much research has focused on the ecological and behavioural effects of habitat complexity on fish (Brown and Day 2002; Spiliopoulos et al. 2025), our understanding of its impact on fish cognition, particularly in the wild, remains scarce (Salena et al. 2021).

**Fig. 1:**
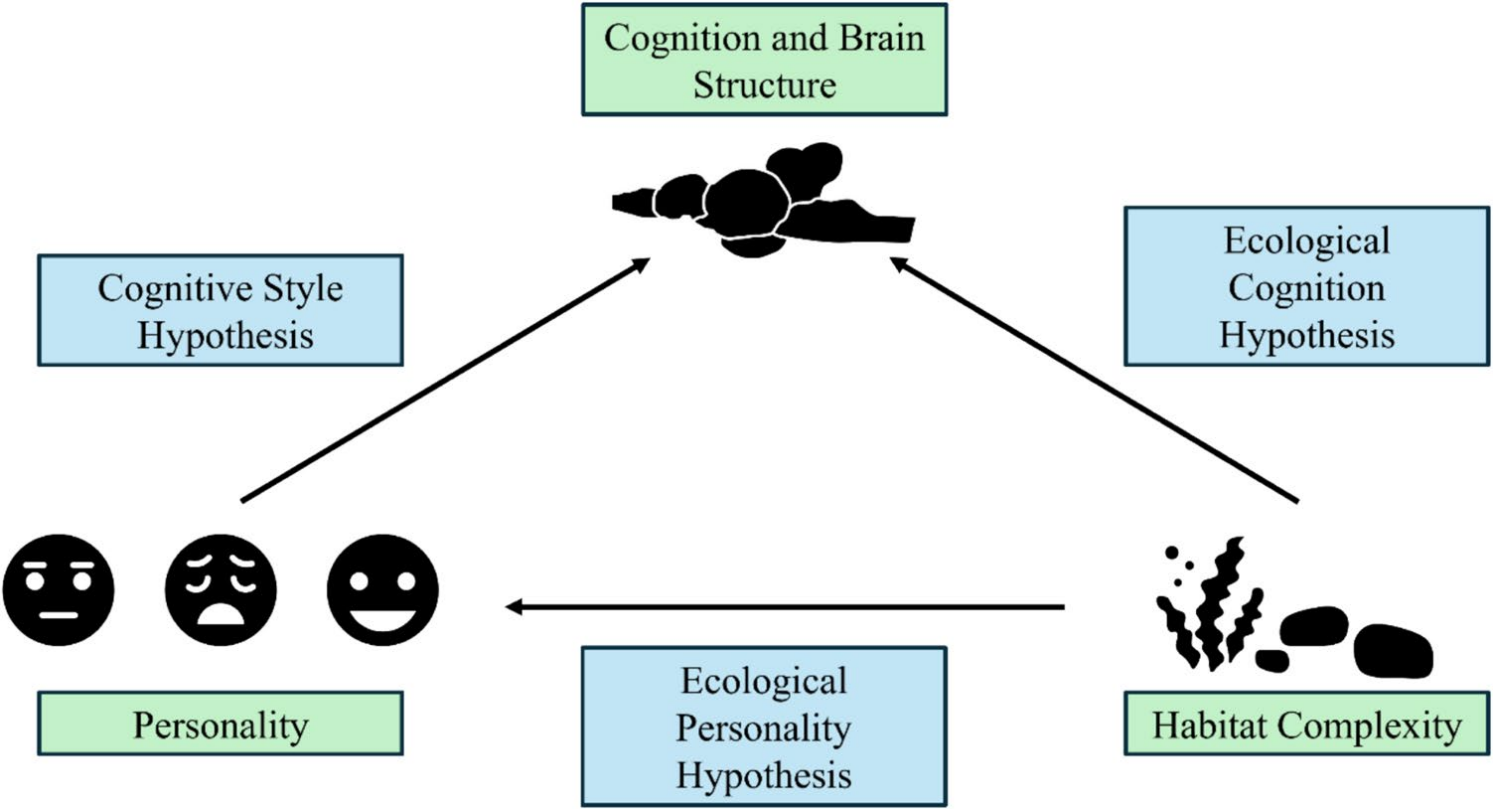
Conceptual overview of the three-way interaction between habitat complexity, personality, and cognition in animals. Arrows indicate the proposed direction of influence. Note that the term *Ecological Personality Hypothesis* is not an established theory but is used here as a conceptual label

Building on this, one of the emerging themes in the study of animal cognition is the interplay with personality. Personality is defined as individual differences in behaviour that are consistent across time and context (Gosling 2001). Several personality traits, such as boldness and activity, can have profound influences on how animals interact with the world around them, including in the context of a test apparatus. For example, bold fish might approach and interact with novel objects more quickly and hence enhance their learning speed. This interaction seems responsible for creating different learning styles (Riding 1997). Personality may also be shaped by habitat complexity, thus there is likely an intricate three-way interaction between personality, habitat complexity, and cognition. Importantly, cognition may be indirectly influenced by habitat complexity via shifts in personality (see Fig. 1).

The objective of this narrative review is to provide a comprehensive synthesis of current knowledge on how the decline of habitat complexity influences fish cognition, brain structure, and personality traits (refer to the Supplementary Methods for information on our methodological approach). We will explore how habitat complexity shapes various cognitive abilities, affects underlying brain structure and specific brain regions, and interacts with fish personality traits. Note that from here on, the terms habitat complexity and environmental enrichment refer specifically to physical structures, rather than other forms of enrichment (e.g., social, sensory, nutritional, or occupational), unless stated otherwise. The review is divided into five parts: First, we examine how habitat complexity shapes brain structure between and within species, while the second part explores how cognitive abilities are impacted between and within species due to habitat complexity. Third, we will address how habitat complexity correlates with personality traits within species. Fourth, we suggest an intricate three-way interaction between habitat complexity, personality, and cognition in fish, whereby changes in habitat complexity can indirectly alter cognition via changes in personality. Finally, we offer a future outlook on how ongoing habitat simplification may shape fish cognition in an increasingly altered world.

## How does habitat complexity shape fish brain structure?

**Fig. 2:**
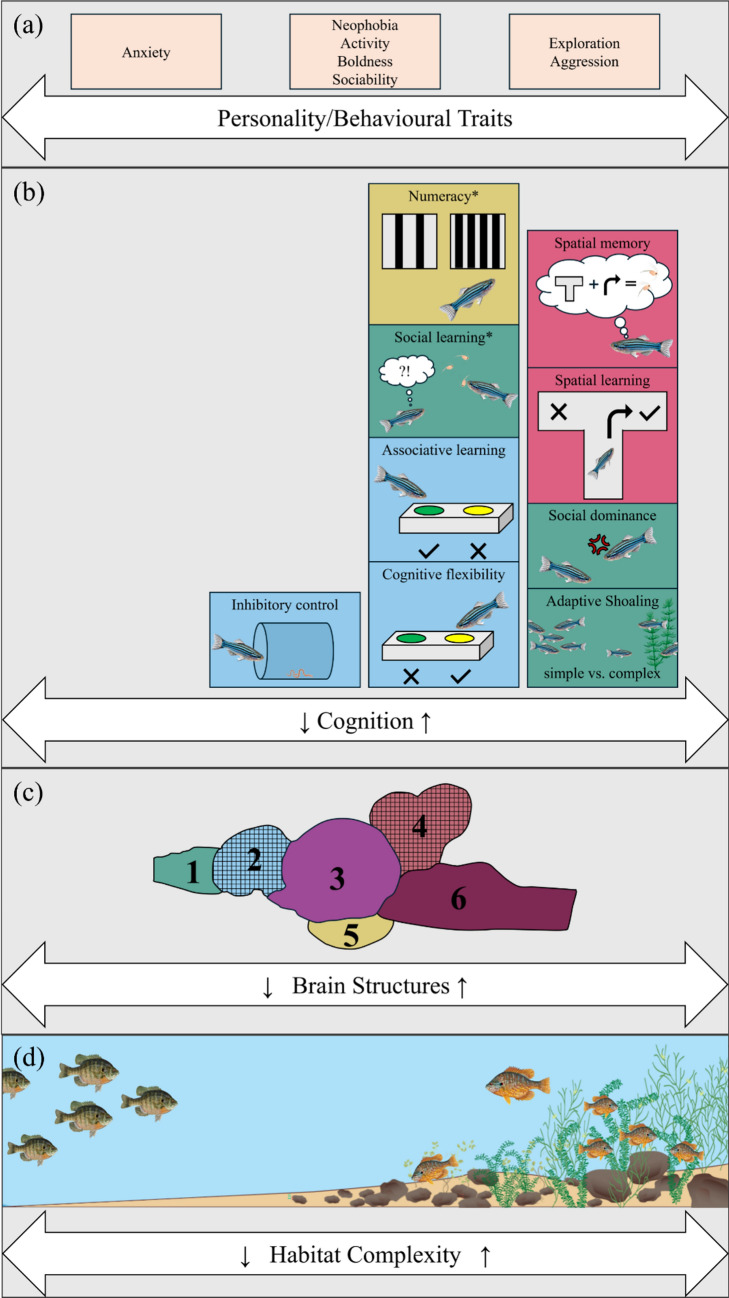
Conceptual overview of the relationship between habitat complexity and (**a**) personality/behavioural traits, (**b**) cognition, and (**c**) brain structures. (**a**) Personality/behavioural traits such as exploration and aggression tend to increase with habitat complexity, whereas low complexity is associated with anxious behaviour. Effects on neophobia, boldness, sociability, and activity are more variable and species dependent. (**b**) Cognitive domains reviewed include spatial cognition (red), general cognition (blue), social cognition (green), and numeracy (yellow). Spatial and general cognition appear to be enhanced by greater habitat complexity. Asterisks (*) denote areas supported by only one study. (**c**) Brain regions shown are: (**1**) olfactory bulb, (**2**) telencephalon, (**3**) optic tectum, (**4**) cerebellum, (**5**) hypothalamus, and (**6**) medulla (redrawn from a walleye brain; Edmunds et al. 2016). Grids indicate regions positively correlated with habitat complexity across species. Relative brain size also tends to increase with habitat complexity. (**d**) Schematic representation of a gradual increase in habitat complexity

Fishes are the most diverse paraphyletic assemblage within vertebrates, comprising around 37 000 species, thus accounting for more than half of all living vertebrate species (Lévêque et al. 2008; Helfman et al. 2009; Burton and Burton 2017). This remarkable diversity is attributed to their adaptation to a wide array of aquatic environments, including extreme habitats such as hypersaline or acidic lakes (Satake et al. 1995; Gonzalez 2012), the freezing temperatures of polar waters (Verde et al. 2006), periods of droughts in deserts (Brown 2013), the high-pressure depths of the deep sea (Cocker 1978), and the perpetual darkness of caves (Poulson 1963). Different selection pressures in these habitats have led to diverse morphological (Su et al. 2019; Brosse et al. 2021) and physiological traits (Schulte and Healy 2022) as well as specialised sensory abilities and behaviours, which can be reflected in brain structure (Kotrschal et al. 1998; Braithwaite 2006; Ullmann et al. 2010). Indeed, fish, as a paraphyletic group, exhibit the greatest variation in brain morphology and function among all vertebrates (Nieuwenhuys et al. 1998). While brain size generally correlates with allometric growth rates (Ridet and Bauchot 1990), the environment also plays a crucial role in shaping brain size and morphology. The Ecological Cognition Hypothesis suggests that an individual’s brain and behaviour are primarily shaped by the environment they inhabit and the challenges encountered throughout their life (Real 1993) (see Fig. 1). Despite this great diversity in fish brain morphology, they closely resemble other vertebrate brains. For instance, their external anatomy reveals that fish share the same major brain divisions as most vertebrates (Northcutt 2002). Additionally, the functions of various brain regions in the fish nervous system are often homologous to those of other vertebrates (Wullimann and Mueller 2004; Broglio et al. 2011). In this narrative review, we will focus mainly on relative brain size as well as six major brain regions that control specific cognitive functions: the olfactory bulbs, telencephalon, optic tectum, medulla, cerebellum, and hypothalamus (see Fig. 2c). Relative brain size refers to the relationship between the size of an animal’s brain and their overall body size, allowing us to compare brains between different species (Striedter 2005). In addition to morphological changes in brain anatomy, we also touch upon the impact of habitat complexity on structural, physiological, or molecular changes in fish brains.

In terms of functionality, these six brain regions show clear signs of homology with the brain regions of other vertebrates (Ullmann et al. 2010). The forebrain in fish consists of the olfactory bulbs and the telencephalon. The olfactory bulbs, occurring as paired structures at the rostral end of the fish brain, are necessary for processing smells. They receive chemosensory input from olfactory receptor neurons located in the olfactory rosettes at the base of the nasal cavity (Laberge and Hara 2001; Kasumyan 2004), which is then transmitted as olfactory information to the telencephalon via projection neurons (Becerra et al. 1994). Hence, the olfactory bulbs play a crucial role in behaviours involved in understanding social cues, such as necessary for reproduction and kin recognition (Døving 2007; Simões et al. 2015), feeding (Laberge and Hara 2001; Yang et al. 2024), predator avoidance (Fischer et al. 2017; Wagner et al. 2023), and prey detection (Hara 1986). In addition to olfactory information, the telencephalon also receives input from the gustatory, auditory, motor, and lateral line centres (van Staaden et al. 1994). The telencephalon is the primary site of higher-order integrative brain functions (Davis et al. 1981; Davis and Kassel 1983). It plays a key role in learning and memory in fish and is involved in complex behaviours linked to avoidance conditioning (Portavella et al. 2003, 2004), spatial navigation (Salas et al. 1996; López et al. 2000), social interactions like territoriality and schooling (Demski and Beaver 2001; Shinozuka and Watanabe 2004), and reproductive strategies including courtship, spawning, nest building, and parental care (Overmier and Gross 1974; Koyama et al. 1984; Pollen et al. 2007; Broglio et al. 2011). Recent studies propose that the telencephalon is the executive brain region in fish, responsible for cognitive flexibility, inhibitory control, and memory permanence (Triki et al. 2022, 2023).

The optic tectum is located in the midbrain of fish. The outer layers of the optic tectum are a primary visual structure, receiving visual information directly from the retinal ganglion cells of the eye via the optic nerve, while the deeper layers are a multimodal structure, receiving sensory inputs from multiple sensory modalities (Stein and Meredith 1993; Sparks 2002; Edmunds et al. 2016). This organisation provides an egocentric framework for salient stimuli, crucial for responding appropriately to the surrounding environment (Northcutt 1983). Additionally, the midbrain includes the hypothalamus, the primary centre of neuroendocrine regulation. The hypothalamus regulates the release of hormones involved in reproduction and stress responses by signalling the pituitary gland (Peter and Fryer 1983; Butler and Hodos 2005), and coordinates daily cycles in physiological state (Adamantidis and de Lecea 2008) as well as food intake (Roberts and Savage 1978), receiving olfactory impulses from the forebrain (Schnitzlein 1964).

The control centre of movements in fish is the cerebellum. It is located in the hindbrain and is responsible for motor activity, posture, locomotion, and other motor-associated behaviours (Bauchot et al. 1977; New 2001). Furthermore, the cerebellum, next to the telencephalon, plays a crucial role in learning, memory, and spatial cognition (Rodríguez et al. 2005; Durán et al. 2014). Last, the medulla, also part of the hindbrain in fishes, is the relay centre for numerous nerves that link the hindbrain with mid- and forebrain (White and Brown 2015b). Regulating blood pressure, heart rate, digestion, and waste disposal, the dorsal medulla, in particular, controls operations of the inner organs and processes taste information, influencing feeding preferences (Huber et al. 1997; Pollen et al. 2007).

### The impact of habitat complexity on brain structure evolution between species

Like relative brain size, different brain regions can greatly differ in relative size and form between fish species to meet the specific demands of their environment (Shumway 2008; Lecchini et al. 2014). The production, maintenance, and operation of brain tissue are energetically costly, prompting animals to invest selectively in specific brain regions based on ecological conditions (Aiello and Wheeler 1995; Dukas 1999; Isler and Van Schaik 2006, 2009; Niven and Laughlin 2008). There is compelling evidence that, in particular, habitat complexity exerts certain cognitive demands across fish species, shaping their brain evolution (Gonzalez-Voyer and Kolm 2010; White and Brown 2015b). To gain a better understanding of the role habitat complexity plays in shaping brain evolution in fish, a common practice is to compare closely related species that inhabit a diverse range of habitats (see Table 1).

**Table 1:** Studies examining the effects of habitat complexity on brain structure in fish, both between (B) and within (W) species. Arrows indicate the direction of the relationship: positive (↑), negative (↓), or inconclusive (→). A cross (×) denotes no effect. Differentially expressed genes and DNA methylation changes are not assigned symbols, as these were not quantified as increases or decreases

Level	Family	Species	Complexity type (high ~ low)	Brain structure ~ Habitat complexity	Reference
B	Alosidae Centrarchidae Esocidae Gobiidae Leuciscidae Lotidae Osmeridae Percidae Percopsidae Salmonidae	16 species	littoral vs. pelagic	telencephalon ↑ cerebellum ↓	Edmunds et al. 2016
B	22 chondrichthyan families	46 shark species 2 holocephalan species	reef vs. benthopelagic	relative brain size ↑ telencephalon ↑	Yopak et al. 2007
B	45 chondrichthyan families	151 species	reef vs. benthopelagic	relative brain size ↑ telencephalon ↑ cerebellum ↑	Yopak et al. 2012
B	36 chondrichthyan families	100 species	reef vs. deep sea	relative brain size ↑ telencephalon ↑ cerebellum ↑ medulla ↓	Mull et al. 2020
B	Centrarchidae	*Lepomis gibbosus * *L. macrochirus*	littoral vs. pelagic	cerebellum ↑	Axelrod et al. 2021
B	Cichlidae	189 species	vegetation/rocks vs. sand/mud vs. pelagic	telencephalon ↑	van Staaden et al. 1994
B	Cichlidae	189 species	vegetation/rocks vs. sand/mud vs. pelagic	relative brain size ↑ telencephalon ↑	Huber et al. 1997
B	Cichlidae	*Xenotilapia ochrogenys * *X. bathyphila * *X. boulengeri * *X. flavipinnis * *X. spiloptera * *Asprotilapia leptura * *Enantiopus melanogenys*	vegetation/rocks vs. sand/mud vs. pelagic	relative brain size ↑ telencephalon ↑ cerebellum ↑ medulla ↓ olfactory bulb ↓	Pollen et al. 2007
B	Cichlidae	35 species	rock vs. sand	relative brain size ↑ telencephalon ↑ cerebellum ↑ medulla ↓ olfactory bulb ↓	Shumway 2008
B	Cichlidae	35 species	rock vs. sand	relative brain size ↑ telencephalon ↑ cerebellum ↑	Shumway 2010
B	Cichlidae	43 species	rock vs. benthopelagic	telencephalon ↑ cerebellum ↑ olfactory bulb ↓	Gonzalez-Voyer and Kolm 2010
B	Eleotrididae Gobiidae Kraemeriidae Microdesmidae Rhyacichthyidae Xenisthmidae	180 species	marine/river vs. mud	relative brain size ↑	Bauchot et al. 1989
B	Gobiidae	*Bathygobius cocosensis * *B. krefftii * *Favonigobius lentiginosus * *Istigobius hoesei*	rockpool vs. sand	relative brain size ↑ telencephalon ↑ optic tectum ↓ hypothalamus ↓	White and Brown 2015b
B	Gobiidae	*Favonigobius gymnauchen * *Istigobius hoshinonis* *Tridentiger trigonocephalus* *Chaenogobius annularis*	rock vs. sand	telencephalon ↑	Yoshida et al. 2020
W	35 teleostean families	113 species	reef vs. benthopelagic	relative brain size ↑	Bauchot et al. 1977
W	Alosidae Centrarchidae Esocidae Gobiidae Leuciscidae Lotidae Osmeridae Percidae Percopsidae Salmonidae	16 species	littoral vs. pelagic	brain regions x	Edmunds et al. 2016
W	Centrarchidae	*Lepomis gibbosus*	littoral vs. pelagic	relative brain size ↑	Axelrod et al. 2018
W	Centrarchidae	*L. gibbosus * *L. macrochirus*	littoral vs. pelagic	relative brain size ↑ telencephalon ↓	Axelrod et al. 2021
W	Cichlidae	*Altolamprologus compressiceps * *Eretmodus cyanosticus * *Interochromis loocki * *Julidochromis ornatus * *Lepidolamprologus elongatus * *Neolamprologus pulcher * *N. tetracanthus * *Telmatochromis temporalis * *Tropheus moorii * *Variabilichromis moorii*	rock vs. sand	relative brain size ↓ cerebellum ↓	Ma et al. 2024
W	Danionidae	*Danio rerio*	plants/gravel vs. barren tanks	brain cell proliferation in telencephalon ↑	von Krogh et al. 2010
W	Danionidae	*Danio rerio*	plants/rocks/sand vs. barren tanks	differentially expressed genes *pcna, neurod, cart4, cnrl* ↓ *nr3c2/nr3c1(α), nr3c1(β)/nr3c1(α)* ↑	Manuel et al. 2015
W	Danionidae	*Danio rerio*	plants/gravel/novel object/shelter vs. barren tanks	relative brain size ↑	DePasquale et al. 2016
W	Danionidae	*Danio rerio*	plants/colourful marbles and pebbles/pipes/bubbles vs. barren tanks	molecular layer volume of cerebellar crest (CCe) x c-Fos immunoreactive cells in CCe x neuronal population in CCe x	Flores-Prieto et al. 2024
W	Gasterosteidae	*Gasterosteus aculeatus*	benthic vs. limnetic	telencephalon ↓	Park and Bell 2010
W	Gasterosteidae	*Gasterosteus aculeatus*	plants/pebbles/pipe vs. barren tanks	relative brain size x brain regions x	Toli et al. 2017
W	Gasterosteidae	*Gasterosteus aculeatus*	vegetation/rocks/other structures vs. mud/sand	relative brain size x brain regions x	Ahmed et al. 2017
W	Gasterosteidae	*Pungitius pungitius*	marine vs. pond	telencephalon ↑ olfactory bulb ↑	Gonda et al. 2009
W	Hypopomidae	*Brachyhypopomus gauderio*	natural setting vs. semi-natural outdoor ponds vs. isolated in barren tanks	brain cell proliferation ↑	Dunlap et al. 2011
W	Poeciliidae	*Gambusia hubbsi*	mangrove/rock vs. mud	cerebellum ↑ optic tectum ↑	Jenkins et al. 2021
W	Poeciliidae	*Poecilia formosa*	pipes/gravel vs. barren tanks	cerebellum ↑	Nadema et al. 2025
W	Poeciliidae	*Poecilia reticulata*	wild-caught vs. lab-reared	relative brain size x brain regions x	Burns et al. 2009
W	Pomacentridae	*Abudefduf saxatilis*	plants/pipes vs. barren tanks	differentially expressed genes (only under simulated ocean warming and habitat simplification)	Swank et al. 2025
W	Rivulidae	*Kryptolebias marmoratus*	plants/shelter vs. barren tanks	DNA methylation changes in brain	Berbel-Filho et al. 2019
W	Rivulidae	*Kryptolebias marmoratus*	plants/shelter vs. barren tanks	DNA methylation changes in brain across generations	Berbel-Filho et al. 2020
W	Salmonidae	*Oncorhynchus kisutch*	cinder blocks/gravel vs. barren/hydrodynamic tanks	brain cell proliferation in telencephalon zone 2 ↓	Lema et al. 2005
W	Salmonidae	*Oncorhynchus kisutch*	semi-natural stream vs. barren hatchery	telencephalon ↑ optic tectum ↓	Kotrschal et al. 2012
W	Salmonidae	*Oncorhynchus kisutch*	wild-caught vs. hatchery-reared	DNA methylation changes in brain	Le Luyer et al. 2017
W	Salmonidae	*Oncorhynchus mykiss*	rocks vs barren tanks	relative brain size ↑ cerebellum ↑	Kihslinger and Nevitt 2006
W	Salmonidae	*Oncorhynchus mykiss*	near-natural streams vs. barren hatchery	DNA methylation changes in brain	Gavery et al. 2019
W	Salmonidae	*Oncorhynchus mykiss*	substrate vs. barren tanks	DNA methylation changes in brain	Reiser et al. 2021
W	Salmonidae	*Oncorhynchus mykiss*	plants/rocks/pipes vs. barren tanks	differentially expressed genes *npas4b, pcna, ncam, neurabin-1 n, tkr2b, stxbp5, stx1b, mapk1, mapk/erk, mtor, creb, dat, vmat2, gbrl2* ↑ *cfos* x	Cardona et al. 2022
W	Salmonidae	*Oncorhynchus tshawytscha*	denuded trees/floating structures vs. barren hatchery	relative brain size x brain regions x	Kihslinger et al. 2006
W	Salmonidae	*Salmo trutta*	streams vs. lake vs. barren hatchery	relative brain size x brain regions x	Závorka et al. 2022
W	Salmonidae	*Salmo salar*	rocks vs. barren hatchery	relative brain size ↑ telencephalon ↑ cerebellum ↑ olfactory bulb ↑ optic tectum ↑	Näslund et al. 2012
W	Salmonidae	*Salmo salar*	pebbles/cobbles/vertical structures vs. barren hatchery	NeuroD1 mRNA expression ↑	Salvanes et al. 2013
W	Salmonidae	*Salmo salar*	gravel vs. barren tanks	808 differentially expressed genes	Evans et al. 2015
W	Salmonidae	*Salmo salar*	plants/rocks vs. barren hatchery	*bdnf* expression in ventral dorsolateral pallium* ↓ * *cfos* x	Mes et al. 2019
W	Scorpaenidae	*Sebastes schlegelii*	plants/other structures vs. barren tanks	cerebellum →	Zhang et al. 2019
W	Scorpaenidae	*Sebastes schlegelii*	plants/other structures vs. barren tanks	differentially expressed genes *fn, pai1, col* ↑	Zhang et al. 2021
W	Scorpaenidae	*Sebastes schlegelii*	plants/other structures vs. barren tanks	brain-derived neurotrophic factor in telencephalon ↑ nerve growth factor in telencephalon ↑	Shen et al. 2023
W	Serrasalmidae	*Colossoma macropomum*	plants/shelter/waterflow vs. barren tanks	brain cell proliferation in telencephalon ↑ 64 differentially expressed genes *ppp2r1b, ppp3r1, msk1*↑	Pereira et al. 2020
W	Sparidae	*Sparus aurata*	plant-fibre ropes vs. barren tanks	antioxidant activity in brain ↑ dopaminergic activity in telencephalon ↑ serotonergic activity in cerebellum ↑	Arechavala‐Lopez et al. 2020

One of the most prominent examples of brain evolution in fish is the African cichlids. They are an excellent and widely used model to study brain structure evolution in vertebrates because of their rapid radiation in the three largest African Lakes (Lake Malawi, Lake Tanganyika, and Lake Victoria), resulting in hundreds of new species in a relatively short evolutionary time (van Staaden et al. 1994; Huber et al. 1997; Pollen et al. 2007). These lakes feature a range of microhabitats that differ in terms of habitat complexity; ranging from simple benthic habitats characterized by sand and mud to rather complex habitats dominated by rubble, rocks, aquatic plants, and reeds (Pollen et al. 2007). Cichlids evolved relatively larger brains when inhabiting reeds and rocky crevices and intermediate-sized brains when occupying sandy and muddy microhabitats, while pelagic species displayed relatively small brains (Huber et al. 1997). In addition to relative brain size, several studies also discovered a positive correlation between habitat complexity and the telencephalon and cerebellum in cichlids (van Staaden et al. 1994; Huber et al. 1997; Pollen et al. 2007; Shumway 2008, 2010; Gonzalez-Voyer and Kolm 2010), possibly enhancing spatial memory, spatial orientation, and visual acuity to meet the ecological demands of more complex habitats (Broglio et al. 2003; Shumway 2010). Pollen et al. (2007) point out that the size of the brain and cerebellum in cichlids was positively correlated with the number of species present in the habitat, which, in turn, is positively correlated with habitat complexity. In contrast, the medulla and olfactory bulbs were negatively correlated with habitat complexity (Pollen et al. 2007; Shumway 2008; Gonzalez-Voyer and Kolm 2010), suggesting a reduced reliance on olfactory cues in complex habitats.

Similar results were observed in chondrichthyans, where species inhabiting complex environments evolved larger relative brains, telencephala, and cerebella (Yopak et al. 2007; Yopak 2012; Mull et al. 2020). For instance, across 151 shark and ray species, carcharhiniform sharks have the largest brains, larger than average telencephala, and higher cerebellar foliation (Yopak et al. 2007; Yopak 2012). Many of these species dwell in or are associated with structurally complex reef environments, including some coastal-pelagic species. Mull et al. (2020) suggest that the concurrent trend of increased brain regions (i.e., telencephalon, cerebellum) responsible for advanced cognitive and motor functions together with total brain size, may have equipped sharks to radiate from deep to shallow, complex habitats, allowing them to capitalize on new, ecological niches. Alongside an increase in relative brain, telencephalon, and cerebellum size, chondrichthyans inhabiting shallow water, coastal, and reef environments also possess relatively larger optic tecta compared to deepwater species, whose brains are dominated by the medulla and olfactory bulbs, suggesting a shift from nonvisual senses in the dark toward a more active visual lifestyle requiring spatial learning (Mull et al. 2020).

In gobies, enlarged brains and telencephala were also associated with species occupying more structurally complex habitats (Bauchot et al. 1977, 1989; White and Brown 2015a; Yoshida et al. 2020). Yet, White and Brown (2015a) reported a negative correlation between relative optic tecta and hypothalamus size with habitat complexity in rock pool- and sand-dwelling gobies. While the optic tectum was the largest brain structure in all four goby species, accounting for approx. 29% of the total brain volume, sand-dwelling gobies had relatively larger optic tecta and hypothalami, likely due to a reduced need for spatial learning in their spatially simple habitats, and a greater need for visual acuity compared to rock pool species.

In contrast with research showing an association between larger cerebella and high habitat complexity in fish, Edmunds et al. (2016) report larger cerebella in freshwater fishes occupying pelagic rather than more structurally complex littoral habitats in Lake Huron in Canada. They hypothesised that the larger cerebellum allowed pelagic species to better navigate the three-dimensional nature of large open waters, especially during predator–prey interactions, as observed in highly active pelagic elasmobranchs (Kruska 1988; Lisney et al. 2007; Edmunds et al. 2016). In contrast, Axelrod et al. (2021) discovered that littoral pumpkinseed sunfish have relatively larger cerebella than pelagic bluegill sunfish because foraging for cryptic macroinvertebrates in structurally complex habitats may require greater motor control and function than feeding on pelagic zooplankton in the open water.

### The impact of habitat complexity on brain morphology within species

There is wide-spread evidence across the animal kingdom that ecological, life-history, and behavioural traits correlate with inter- and intra-specific variation in brain structure (Ito et al. 2007). Fish are renowned for their incredible capacity for neural plasticity which lasts throughout ontogeny (Zupanc 2002). Because of this, they rapidly adjust both brains and behaviour to match contemporary conditions, often generating population-level variation. Wild-caught ninespine sticklebacks collected from a range of environments (i.e., marine, lake, or pond habitats), for example, showed population variation in brain size as well as the relative size of various lobes (Gonda et al. 2009, 2011). Studies on threespined sticklebacks also showed significant variation in telencephalon size and shape in response to variation in ecology (Park and Bell 2010), and work on gambusia found variation in telencephala in response to anthropogenic impacts—differences in the degree of habitat fragmentation—as well as larger cerebella and optic tecta in populations from sites with greater structural complexity (Jenkins et al. 2021).

With regard to habitat complexity, there is a clear trend in brain morphology variation between species, with fish species from structurally complex habitats typically showing larger relative brains, telencephala, and cerebella (see Fig. 2c). However, we don’t observe the same effect within species; instead, habitat complexity seems to drive species-specific brain morphology adaptations. For instance, in pumpkinseed sunfish, individuals inhabiting the structurally complex littoral zone had 8.3% larger brains than pelagic individuals (Axelrod et al. 2018). Yet, the same effect was not observed for bluegill sunfish ecotypes. Here, pelagic bluegill displayed larger relative telencephala compared to their littoral ecotypes, suggesting increased cognitive requirements to navigate the three-dimensional, open environment of the pelagic for foraging (Park and Bell 2010; Gonda et al. 2011) and the necessity of increased spatial learning and memory to move from the pelagic to the littoral zone for spawning (Axelrod et al. 2021). In African cichlids, brain morphology variation in correlation with habitat complexity within species contradicts the findings of previous work among species. While empirical evidence suggests that there is an increase in relative brain size in species from structurally complex habitats (Huber et al. 1997; Pollen et al. 2007; Shumway 2008), a recent study discovered a negative association between habitat complexity and brain size within cichlid species. Specifically, individuals from low complexity habitats had up to 27.06% larger brains and disproportionately larger cerebella by 12.56% compared to individuals from more complex habitats (Ma et al. 2024). In contrast, other studies did not find any effect of habitat complexity on brain morphology variation within species in the wild (Edmunds et al. 2016; Ahmed et al. 2017).

While natural intra-specific population variation in brain morphology seems to be ubiquitous in fishes, artificial environments can also generate variation as part of a broader domestication syndrome. Studies conducted on rainbow trout, for example, showed that the brains of hatchery-reared fish are substantially smaller across multiple areas relative to wild fish (Marchetti and Nevitt 2003). Much of this decline in brain size in hatcheries is due to the lack of physical structures in their rearing environment (perhaps also the social environment and stressors) and can be rescued to some extent through physical environmental enrichment. Multiple studies showed that physical enrichment in hatcheries ultimately leads to enhanced brain size (Kihslinger and Nevitt 2006; Näslund et al. 2012; DePasquale et al. 2016). As an example, Coho salmon reared in near-natural streams rather than under barren hatchery conditions developed significantly larger telencephala but smaller optic tecta (Kotrschal et al. 2012), and steelhead salmon grew significantly larger cerebella and larger brains under structurally complex rearing conditions (Kihslinger and Nevitt 2006). Furthermore, Atlantic salmon alevins raised in structurally enriched trays grew larger brains and brain substructures compared to alevins raised under barren conditions, yet these effects disappeared over time once fish were transferred to barren, feeding tanks (Näslund et al. 2012). These rapid shifts are indicative of the tremendous capacity for plasticity in fish brains. It is important to note, however, that these positive effects of physical enrichment are not universal. In some cases, no effect on brain structure was observed (Kihslinger et al. 2006; Burns et al. 2009; Toli et al. 2017; Závorka et al. 2022).

### The impact of habitat complexity on brain structure and gene expression within species

In addition to morphological changes in brain structure, habitat complexity can also lead to physiological and molecular changes as well as changes in neural connectivity in fish brains. Studies manipulating habitat structure have illuminated some of the underlying causal mechanisms. In zebrafish, for example, rearing fish in a physically enriched environment for just a week increased cell proliferation in the telencephalon as indicated by immunohistochemistry (von Krogh et al. 2010). Similarly, weakly electric bluntnose knifefish showed greater rates of brain cell proliferation in individuals from a natural, complex environment compared to conspecifics reared in semi-natural outdoor ponds or isolated, barren tanks (Dunlap et al. 2011). In contrast, one study found a negative correlation between brain cell proliferation in the telencephalon and habitat complexity in coho salmon, possibly because fish from the high complexity treatment were less active due to a lower flow velocity in these tanks (Lema et al. 2005). These shifts in neural tissue are also underlined by changes in gene expression (Salvanes et al. 2013; Evans et al. 2015; Zhang et al. 2021; Cardona et al. 2022).

A few studies have even identified alterations in the expression of genes associated with cognition and brain morphology in fish exposed to increased habitat complexity. Moreover, epigenetic modifications, such as genome-wide changes in DNA methylation, have been observed in hatchery versus wild-reared fish and in fish reared with or without physical enrichment, suggesting a regulatory mechanism linking environment to gene expression (Le Luyer et al. 2017; Gavery et al. 2019; Reiser et al. 2021). Salvanes et al. (2013) found that Atlantic salmon reared in physically enriched environments had enhanced expression of the transcription factor NeuroD1 in the telencephalon, a gene linked to neurogenesis. Another study on Atlantic salmon found that physical enrichment caused a lower expression of *bdnf* in the ventral dorsolateral pallium, a gene promoting neural remodelling (Mes et al. 2019). Mes et al. (2019) hypothesised that the decrease in *bdnf* expression indicates stress-alleviating effects of habitat complexity in salmon. In rainbow trout, physical enrichment significantly enhanced the expression of genes related to cerebral activity (*dat, vmat2, gabr7l2, chat*), neural plasticity (*mtor, npas4b, ntrk2b, mapk1, creb, neurabin-1*), neurogenesis (*pcna*), and synaptogenesis (*ncam, stxbp5, ntrk2b, mapk1, neurabin-1*) in the telencephalon (Cardona et al. 2022). In particular, the genes *mtor* and *creb* have been associated with long-term memory (Yin and Tully 1996; Ciccarelli and Giustetto 2014; Peixoto et al. 2017), and the *mapk*/*erk* pathway plays a critical role in brain development, learning, and memory (Samuels et al. 2009). Increased habitat complexity also induced significant beneficial effects on the expression of *ppp2r1b*, *ppp3r1*, and *msk1* in tambaqui (Pereira et al. 2020), genes playing a key role in learning and memory, and cognitive enhancement (Karelina et al. 2012; Shioda et al. 2017).

It is worth noting that some studies also found contradicting results. In an inhibitory avoidance experiment, Manuel et al. (2015) found that 6-month-old zebrafish raised in physically enriched environments showed reduced expression of *pcna, neurod, cart4*, and *cnr1*, genes associated with cognitive plasticity. The downregulation of these genes was in line with the also observed decrease in aversive learning performances in fish from the high complexity treatment. In a coral reef fish, the sergeant major, the loss of habitat complexity alone did not affect gene expression significantly, but if combined with heat stress, the expression of genes associated with synaptic plasticity and spatial memory was significantly altered (Swank et al. 2025). Moreover, several studies found no effect of habitat complexity on the neural activity marker *cfos* (Mes et al. 2019; Cardona et al. 2022; Flores-Prieto et al. 2024), usually associated with brain processing capacity.

### Impact of habitat complexity on brain structure; intergenerational and transgenerational effects

Interestingly, the influence of environmental enrichment may transcend generations via epigenetic mechanisms. This can manifest in a variety of guises, including parental care, investment in oocytes (size and quality), and transfer of stress hormones (McGhee and Bell 2014). Parental effects have important ecological and evolutionary consequences (Reznick 1991). As physical environmental enrichment can also have profound effects on DNA methylation patterns across the whole genome, including in the brain (e.g., rainbow trout; Reiser et al. 2021), it seems reasonable to assume that some of these effects may be passed on to offspring. Studies on zebrafish, for example, show that parental and even grandparental exposure to varying levels of habitat complexity can alter both behaviour and morphology of offspring (Green and Swaney 2023). Experiments examining epigenetic impacts of habitat complexity on brain structures are lacking; nonetheless, studies on mangrove killifish found that offspring whose parents had been reared in physically enriched environments showed differences in neophobia and activity, as well as 3 brain methylation patterns matching their parents irrespective of the environment they themselves had experienced (Berbel-Filho et al. 2020). Thus, parental environment seems to influence both behaviour and brain methylation patterns in offspring and more studies are needed in the context of habitat complexity.

## Habitat complexity and its effects on fish cognition

### Four main areas of fish cognition research

While the field of fish cognition has a reasonably long history dating back over 100 years, when comparative psychologists studied goldfish alongside other model organisms, including rats and pigeons (Churchill Jr. 1916), fish cognition has historically been overlooked to a certain degree. Only in recent decades has the research interest in fish cognition spiked drastically, mainly due to the growing recognition that fish are intelligent and sentient animals (Brown et al. 2011; Patton and Braithwaite 2015; Sneddon and Brown 2020). As fish are also one of the most utilised and valuable vertebrate taxa to humans, this rise in attention towards fish cognition opened new discussions about the importance of fish welfare, protection, and ethics (Brown 2015). Research on fish cognition has mainly focused on four main areas: general cognition (which includes simple learning and executive functions), spatial cognition, social cognition, and numeracy (Salena et al. 2021).

General cognition describes the ability of animals to perceive and employ information from past experiences to guide future decision-making (Cauchoix and Chaine 2016). This field of research encompasses processes linked to simple learning (i.e., habituation, associative forms of learning, including classical and operant conditioning) as well as executive functions (i.e., cognitive flexibility, inhibitory control, and working memory) (López et al. 2000; Salena et al. 2021; Triki et al. 2023). Cognitive flexibility allows individuals to adapt and change their behaviour in response to shifting environments (Lucon-Xiccato and Bisazza 2017; Uddin 2021; Brunet et al. 2023). Inhibitory control allows individuals to suppress immediate impulses in favour of performing behaviour that will yield greater benefits in the longer term (Miller et al. 2019; Lucon-Xiccato 2024). Finally, working memory becomes important when individuals have to hold temporary information and work with visual-spatial information that is no longer perceptually present (Bloch et al. 2019; Triki et al. 2023; Bonin et al. 2025). General cognition is therefore crucial for an individual’s survival from the earliest life stages onwards, enabling fish to adapt to environmental changes (Kieffer and Colgan 1992; Brown 2001; Valente et al. 2012). Spatial cognition refers to the ability of animals to acquire, process, and reorganize spatial information to understand and navigate an environment (Poucet 1993). The capacity to move around the environment in a directed and efficient manner clearly has fitness benefits. The sensory capabilities of fish allow them to use a multitude of cues for orientation and navigation, such as visual, olfactory, auditory, lateral line, and electrosensory information (Rodríguez et al. 2021), to name a few. Moreover, like mammals and birds, fish can employ allocentric (“external world-centered”) or egocentric (“body-centered”) strategies or both to navigate an environment (Rodriguez et al. 1994; Schluessel and Bleckmann 2005). Allocentric strategies involve the creation of an internal map of spatial relationships among landmarks, allowing fish to locate a place from different directions and adopt novel routes from points previously unvisited (Rodriguez et al. 1994; Jorge et al. 2012; White and Brown 2013). In contrast, egocentric strategies range from taxis, stereotyped stimulus–response associations, and guidance behaviour, based on body-centered frames of spatial reference (López et al. 1999; Fuss et al. 2014; Rodríguez et al. 2021). Spatial cognition plays a key role in many fish behaviours, among other things migration, foraging, predator-avoidance, and mating (Dodson 1988; Markel 1994; Hughes and Blight 1999; Fukumori et al. 2010; Queller et al. 2023). Social cognition enables animals to acquire, process, store, and act on information from other individuals (Zuberbühler and Byrne 2006; Bshary et al. 2014). Social recognition, social learning, inter- and intraspecific cooperation, conflict resolution, and collective decision-making—often regarded as highly complex skills—have all been documented in fish (Brown and Laland 2003; Griffiths 2003; Bshary et al. 2006; Grosenick et al. 2007; Balshine and Buston 2008). Finally, numeracy involves the ability to discern between discrete or continuous quantities (Agrillo et al. 2011). Using quantitative assessments can help individuals to make smart, ecological decisions, leading to increased foraging efficiency, enhanced vigilance, and improved predator defence (Agrillo et al. 2017). Generally, animals use an object filing system (counting) for comparing small numbers or a ratio system that follows Weber’s Law for comparing large quantities. Like many animals, fish struggle to use the object tracking system when groups of objects are larger than 4 or 5 (Agrillo et al. 2008, 2017; Dadda et al. 2009).

Generally, an animal’s ecology and the environment they live in (social and physical) influence their cognition (Pollen et al. 2007; Shumway 2008; Boesch 2021). Habitat complexity, referring to the physical environment, is positively correlated with enhanced cognitive abilities in fish (Shumway 2008; Brown 2012; White and Brown 2015a; Axelrod et al. 2021; Boesch 2021), particularly when it comes to general and spatial cognition.

### Impact of habitat complexity on fish cognition between species

Natural selection seems to operate in a very targeted fashion when it comes to enhancing behaviour to suit specific cognitive requirements rather than acting on general intelligence. Evidence from both birds and mammals, for example, suggest that species that require enhanced spatial learning skills tend to develop larger telencephala (or hippocampi) but learning in other domains remains unchanged (Gaulin and Fitzgerald 1989; Balda and Kamil 2006). In a series of studies, Brown and White examined the differences in cognition and brain morphology in intertidal gobies with the working assumption that fish from complex rockpool environments would likely have better spatial learning capacities than sand dwelling species. Rockpool fish probably need great spatial learning skills to ensure they remain in rockpool refugia at low tide and not get stranded as the tide recedes. An initial study found that rockpool gobies had an astonishing ability to return to their home pools even after being displaced considerable distances (up to 30 m) (White and Brown 2013). These rockpool dwelling species were then compared to sand dwelling species in an artificial rockpool system where fish had to locate a single rockpool of four that provided refuge at low tide. Rockpool gobies located refuge in about 95% of trials while species from sandy shores only succeeded in 10% of cases (White and Brown 2014). The fish were then trained in a t-maze to locate a hidden reward and could use either visual landmarks or turn direction to reliably solve the spatial learning task. Rockpool fish solved the task much faster, made fewer errors and used both cues to orientate within the maze, whereas sand dwelling species overwhelmingly relied on turn direction as their primary orientation cue (White and Brown 2015a). Examinations of brain morphology revealed that rockpool fish had larger brains, and telencephala in particular—an area of the brain long associated with spatial learning (White and Brown 2015b). Thus, the evidence from fishes seems to match both birds and mammals.

### Impact of habitat complexity on fish cognition within species

Several studies have also found evidence for variation within species depending on the habitat they experience during ontogeny, indicative of developmental plasticity. Intertidal gobies, for example, were collected from rockpools and reared in the lab in either complex rockpool, seagrass, or mundane sandy environments. Examination of their spatial learning skills revealed that those maintained in rockpools reached learning criteria faster than those housed in the other two environments (Carbia and Brown 2019). Similar results have been found in Atlantic salmon (Salvanes et al. 2013), rockfish (Zhang et al. 2021; Shen et al. 2023), climbing perch (Sheenaja and Thomas 2011), zebrafish (Spence et al. 2011; Roy and Bhat 2016), barred knifejaw (Makino et al. 2015), rainbow trout (Ahlbeck Bergendahl et al. 2016), tambaqui (Pereira et al. 2020), and gilthead sea bream (Arechavala-Lopez et al. 2020) to name a few (see Table 2; see Fig. 2). Nevertheless, this trend is not universal across species as shown in threespined sticklebacks (Brydges and Braithwaite 2009), guppies (Peña and Bloch 2024), and chinook salmon (Cogliati et al. 2019), where no effect of habitat complexity on spatial cognition was found. Instead, threespined sticklebacks displayed cue-specific spatial learning abilities depending on population origin. For instance, pond populations live in structurally complex habitats with more vegetation and less water movement, causing fish to employ visual cues (landmarks) over egocentric cues (turn left or right) in spatial learning tasks (Girvan and Braithwaite 1998; Odling-Smee et al. 2003; Sheenaja and Thomas 2011; Bensky and Bell 2018). In contrast, river populations depend more on egocentric spatial cues because they experience less structurally complex environments, where visual cues are unlikely to persist over time due to changes in water flow. Another study by Odling-Smee et al. (2008) assessed spatial learning abilities in littoral and pelagic threespined stickleback populations inhabiting several lakes in Canada. They discovered that both populations use visual as well as egocentric cues; however, individuals occupying the structurally more complex littoral zone learnt twice as quickly compared to pelagic individuals.

**Table 2:** Studies examining the effects of habitat complexity on general cognition (G), spatial cognition (Sp), social cognition (So), and numeracy (N) in fish. Arrows indicate the direction of the relationship: positive (↑) or negative (↓). A cross (×) denotes no effect. Note that cue-specific learning under spatial cognition has no symbols assigned because it refers to fish either using egocentric or visual cues in low or high complexity environments, respectively

Level	Family	Species	Complexity type (high ~ low)	Cognition ~ Habitat complexity	Reference
G	Cichlidae	*Neolamprologus pulcher*	rocky vs. rocky/sandy patches	inhibitory control ↓	Jungwirth et al. 2024
G	Danionidae	*Danio rerio*	plants/rocks/sand vs. barren tanks	associative learning ↓	Manuel et al. 2015
G	Danionidae	*Danio rerio*	plants/bamboo sticks/gravel/sand vs. barren tanks	associative learning x cognitive judgement bias x	Buenhombre et al. 2023
G	Gasterosteidae	*Gasterosteus aculeatus*	pond vs. river	associative learning x	Girvan and Braithwaite 1998
G	Gasterosteidae	*Gasterosteus aculeatus*	plants/gravel/shelter vs. gravel tanks	associative learning x cognitive flexibility x	Brydges and Braithwaite 2009
G	Gasterosteidae	*Gasterosteus aculeatus*	changing physical structure vs. barren tanks	inhibitory control ↑	Álvarez-Quintero and Kim 2024
G	Poeciliidae	*Gambusia affinis*	vegetation/sediments vs. urbanized/degraded streams	inhibitory control ↓	Irwin et al. 2024
G	Poeciliidae	*Poecilia reticulata*	plants/gravel vs. barren tanks	associative learning ↑ cognitive flexibility x inhibitory control x	Montalbano et al. 2022
G	Salmonidae	*Oncorhynchus mykiss*	plants/rocks/pipes vs. barren tanks	associative learning ↑ cognitive flexibility ↑ generalization abilities x	Brunet et al. 2023
G	Salmonidae	*Salmo salar*	plants/wood/rocks/pipes/gravel vs. barren tanks	associative learning ↑	Brown et al. 2003
G	Salmonidae	*Salmo salar*	plants/rocks vs. barren hatchery	associative learning ↑	Mes et al. 2019
Sp	Anabantidae	*Anabas testudineus*	stream vs. pond	spatial learning ↑	Sheenaja and Thomas 2011
Sp	Danionidae	*Danio rerio*	plants vs. barren tanks	spatial learning ↑	Spence et al. 2011
Sp	Danionidae	*Danio rerio*	plants vs. barren tanks	spatial learning ↑ spatial memory ↑	Roy and Bhat 2016
Sp	Gasterosteidae	*Gasterosteus aculeatus*	pond vs. river	cue-specific learning	Girvan and Braithwaite 1998
Sp	Gasterosteidae	*Gasterosteus aculeatus*	pond vs. river	cue-specific learning	Odling-Smee and Braithwaite 2003
Sp	Gasterosteidae	*Gasterosteus aculeatus*	benthic vs. limnetic	spatial learning ↑ cue-specific learning	Odling-Smee et al. 2008
Sp	Gasterosteidae	*Gasterosteus aculeatus*	plants/pebbles/plant pots vs. barren tanks	spatial memory x	Brydges and Braithwaite 2009
Sp	Gasterosteidae	*Gasterosteus aculeatus*	vegetation vs. more open/fast flowing water sites	cue-specific learning	Bensky and Bell 2018
Sp	Gobiidae	*Bathygobius cocosensis*	oyster/rockpool vs. sand/seagrass tanks	spatial learning ↑	Carbia and Brown 2019
Sp	Gobiidae	*Favonigobius gymnauchen * *Istigobius hoshinonis * *Tridentiger trigonocephalus * *Chaenogobius annularis*	rockpool vs. sand	spatial memory ↑ visual landmark use ↑	White and Brown 2014
Sp	Gobiidae	*Favonigobius gymnauchen * *Istigobius hoshinonis * *Tridentiger trigonocephalus * *Chaenogobius annularis*	rockpool vs. sand	spatial learning ↑	White and Brown 2015a
Sp	Gobiidae	*Favonigobius gymnauchen * *Istigobius hoshinonis * *Tridentiger trigonocephalus * *Chaenogobius annularis*	rockpool vs. sand	spatial learning ↑	White and Brown 2015b
Sp	Oplegnathidae	*Oplegnathus fasciatus*	seagrass/bricks/tripod structures vs. barren tanks	spatial learning ↑	Makino et al. 2015
Sp	Poeciliidae	*Poecilia reticulata*	plants/gravel vs. barren tanks	spatial learning x	Peña and Bloch 2024
Sp	Salmonidae	*Oncorhynchus mykiss*	plants/shelter/novel objects/pipes/gravel vs. barren tanks	spatial learning ↑ spatial orientation ↑	Ahlbeck Bergendahl et al. 2016
Sp	Salmonidae	*Oncorhynchus tshawytscha*	PVC structures/rocks vs. barren hatchery	spatial learning x	Cogliati et al. 2019
Sp	Salmonidae	*Salmo salar*	pebbles/cobbles/vertical structures vs. barren hatchery	spatial learning ↑	Salvanes et al. 2013
Sp	Scorpaenidae	*Sebastes schlegelii*	plants/other structures vs. barren tanks	spatial learning ↑ spatial memory ↑	Zhang et al. 2021
Sp	Scorpaenidae	*Sebastes schlegelii*	plants/other structures vs. barren tanks	spatial learning ↑	Shen et al. 2023
Sp	Serrasalmidae	*Colossoma macropomum*	plants/shelter/waterflow vs. barren tanks	spatial learning ↑	Pereira et al. 2020
Sp	Sparidae	*Sparus aurata*	plant-fibre ropes vs. barren tanks	spatial learning ↑	Arechavala‐Lopez et al. 2020
So	Gadidae	*Gadus morhua*	kelp/pebbles vs. barren hatchery	adaptive shoaling behaviour ↑	Salvanes et al. 2007
So	Gadidae	*Gadus morhua*	kelp/pebbles vs. barren hatchery	social learning ↑	Strand et al. 2010
So	Leuciscidae	*Phoxinus phoxinus*	boulders/gravel/pebbles vs. gravel/pebbles flow-through tanks	adaptive shoaling behaviour ↑	Orpwood et al. 2008
So	Salmonidae	*Oncorhynchus mykiss*	submerged tree structures/overhead cover vs. barren hatchery	social dominance ↑	Berejikian et al. 2000
So	Salmonidae	*Oncorhynchus mykiss*	submerged tree structures/overhead cover vs. barren hatchery	social dominance ↑	Berejikian et al. 2001
N	Danionidae	*Danio rerio*	colourful Lego bricks vs. barren tanks	number discrimination ↑	Santacà et al. 2024

Habitat complexity can also play a key role in general cognition processes in fish. Associative learning skills, for instance, have been enhanced in Atlantic salmon reared in structurally complex instead barren environments, improving their foraging performance (Brown et al. 2003) and post-release survival (Mes et al. 2019). Moreover, structural complexity led to better performances of rainbow trout in an associative and reversal learning colour discrimination task (Brunet et al. 2023). Yet, rainbow trout from the barren treatment performed equally well in a colour-based generalisation task, suggesting that colour-based generalisation may be a simpler cognitive process than discriminative learning and cognitive flexibility (Brunet et al. 2023). There have been cases which seem to contradict the general pattern. Montalbano et al. (2022), for example, found that guppies reared in barren environments did just as well as those reared in enriched environments in a reversal learning and inhibitory control task, but enriched fish solved the initial colour discrimination task faster as predicted. It is worth noting that the fish reared in the barren environment were also reared in social isolation, in this case, whereas the enriched environments contained both social and physical enrichment. Similarly, experiments where sticklebacks were reared under varying levels of habitat complexity also failed to find differences in associative learning and cognitive flexibility skills (Brydges and Braithwaite 2009). Zebrafish larvae were tested 14 days post fertilisation using an object familiarity test, and results showed that those reared in the barren environment spent more time investigating the familiar object whereas those reared in physically enriched environments examined the familiar and novel object in equal measure (Gatto et al. 2022). It may be that the apparent decline in object recognition may have been due to a reduction in neophobia rather than a shift in learning and memory. Nonetheless, this result suggests that shifts in behaviour and cognition can happen very early in development. Furthermore, 6-month-old zebrafish displayed reduced aversive learning skills when reared under physical enrichment (Manuel et al. 2015). Studies also found mixed trends regarding the link between habitat complexity and inhibitory control abilities in fish. While motor inhibitory control abilities improved in threespined sticklebacks from enriched conditions (Álvarez-Quintero and Kim 2024), the opposite trend was found in western mosquitofish (Irwin et al. 2024) and a Lake Tanganyika cichlid (Jungwirth et al. 2024).

While many studies have examined the effects of habitat complexity on general and spatial cognition, research linking habitat complexity with social cognition and numeracy remains scarce. Nevertheless, it seems that habitat complexity has a significant effect on shoaling behaviour in fish. In European minnows, for instance, shoaling behaviour in the presence of a predator was much more pronounced by forming larger shoals in low complexity habitats, whereas fish from the complex habitats tended to reduce their rates of movement (Orpwood et al. 2008). Furthermore, habitat complexity enhanced adaptive shoaling behaviour in juvenile cods (Salvanes et al. 2007). Juvenile cods from spatially enriched treatments shoaled more tightly in an open area, but reduced shoaling behaviour when a rocky substrate with crevices and shelter opportunities was present. In contrast, juvenile cods from a barren rearing tank displayed shoaling behaviour regardless of the structural complexity of the testing arena, unable to adapt their shoaling behaviour. In rainbow trout, structurally enriched rearing environments led to enhanced competitive abilities in fry and juveniles, enabling them to reach greater social dominance ranks when size-matched with competitors from a conventional hatchery environment (Berejikian et al. 2000, 2001). Next to shoaling behaviour and social interactions, habitat complexity also influenced social learning abilities in fish. For example, naïve juvenile cod raised in structurally complex tanks learned to forage for live prey more efficiently after observing experienced tutors repeatedly, whereas plain-reared fish did not (Strand et al. 2010).

To our knowledge, only one study has so far investigated the effect of habitat complexity on numeracy skills in fish. Santacà et al. (2024) reared zebrafish larvae in either barren or structurally enriched containers and tested their continuous quantity and numerical discrimination skills. To assess continuous discrimination, the authors exploited the innate tendency of zebrafish to navigate an obstacle by passing through the wider opening when at least two options are available. They found that larvae from both treatments solved the continuous quantity task equally well (Santacà et al. 2024). The numerical discrimination task (2 vs. 4 and 2 vs. 3 black stripes on a white background) was based on the observation that zebrafish, like many other animals, prefer environments with vertical stripes, mimicking vegetated habitats (Rimstad et al. 2017; Lucon-Xiccato et al. 2018). Only larvae from the enriched treatment appeared able to discriminate between numericities of 4 and 2, spending significantly more time in the area with more stripes. However, neither group showed evidence of numerical discrimination in the more difficult 2 vs. 3 comparisons (Santacà et al. 2024).

Taken together, studies to date suggest that habitat complexity and the rearing environment can result in shifts in cognition, but the outcome may vary depending on the species, the developmental stage, and the type of enrichment.

## Effects of habitat complexity on fish behaviour and personality

### Personality traits, behavioural syndromes, and cognitive styles

Individual variation in behaviour is the raw material of natural selection. Animal personality traits, behavioural syndromes, and cognitive styles are all aspects of individual differences in animal behaviour, but they each describe a different level or dimension of that consistency. Animal personality traits are consistent, long-term behavioural differences among individuals of the same species over time and or contexts (Gosling 2001). These traits are often measured along a continuum and include traits such as boldness, aggressiveness, sociability, neophobia, activity, anxiety, and exploratory behaviour (Réale et al. 2007; Carter et al. 2013; Gibelli et al. 2019). For example, an individual fish that consistently approaches novel objects more readily than others in its group might be considered a "bolder" and a less “neophobic” individual (Budaev and Brown 2011). It is important to note that many studies examining behavioural variation do not necessarily measure personality traits. As animal personality is a relatively new research concept, many earlier studies focused on behavioural variation per se rather than stable, repeatable individual traits. Strictly speaking, only studies that measured individual behaviours multiple times and calculated among- and within-individual variance to account for behavioural repeatability (R) can be considered a true assessment of animal personality traits (Dingemanse and Wright 2020). Behavioural syndromes are correlated suites of personality traits (Sih et al. 2004) wherein an individual’s behaviour in one context is predictably linked to its behaviour in another. For example, a "proactive" behavioural syndrome might be a suite of traits where an individual is consistently bold, aggressive, and exploratory. In contrast, a "reactive" individual might be consistently shy, non-aggressive, and cautious (Koolhaas et al. 1999; Øverli et al. 2007). Syndromes involving multiple traits suggest that behaviour is not infinitely plastic since each trait is somewhat constrained by the others (Sheehy and Laskowski 2023). Cognitive styles describe consistent individual differences in how an animal processes information (Riding 1997). This can include variations in problem-solving strategies, learning abilities, and decision-making biases. A common framework for cognitive styles is the speed-accuracy trade-off. For example, some juvenile mulloway had a "fast" cognitive style, making quick decisions that may be inaccurate, while others had a "slow" but more accurate style, taking more time to gather information (Raoult et al. 2017). These cognitive styles are often linked to personality traits and behavioural syndromes, where a bold, proactive individual might also have a faster, more risk-prone cognitive style (Carere and Locurto 2011; Sih and Del Giudice 2012a, b) (see Fig. 1).

### Impact of habitat complexity on fish personality within a species

The interplay between habitat complexity and personality within species is multifaceted. On the one hand, habitat complexity may select for different personality traits primarily through phenotypic plasticity (see Fig. 1); conversely, different personality types may actively choose to live in different habitats. For example, desert gobies had divergent bold-exploratory traits associated with their source habitat, displaying a rapid phenotypic response to ecological pressures (Moran et al. 2017). Similarly, a few studies found significant correlations between structural complexity and personality traits in fish. Tracking data of bullhead, for instance, revealed that their relative use of habitat varied with individual aggressiveness; fish associated with complex habitats were less aggressive than those making greater use of open habitats (Kobler et al. 2011). Kobler et al. (2011) hypothesised that fish occupying less structured habitats were more aggressive as the defence of these territories was more challenging in comparison to more structurally complex habitats (see Table 3, see Fig. 2). In contrast, Church and Grant (2019) found that in a two-patch choice experiment, dominant fish clearly preferred complex habitats over open ones, which they aggressively guarded from conspecifics, while subordinate fish stayed mainly in the open habitat. This study is a good example of how different personality types may actively choose to live in different habitats. Interestingly, in the same study, dominant fish refrained from feeding and showed decreased agonistic displays towards conspecifics when placed in an open tank (Church and Grant 2019). However, the authors state that this reticent behaviour was not due to being inherently shy, as dominant fish showed no difference in boldness across contexts. Rather, they suggested that larger, dominant fish protected their higher reproductive value instead of engaging in risky behaviour in the open tank arenas, following the asset protection principle (Church and Grant 2019).

**Table 3:** Studies examining the effects of habitat complexity on personality (P) and behavioural (B) traits in fish. Arrows indicate the direction of the relationship: positive (↑) or negative (↓), or inconclusive (→). A cross (×) denotes no effect or unidirectional trend. The repeatability column indicates if the study measured an individual’s behaviour multiple times across time and/or context (M) or once (O). Studies that measured individual behaviours multiple times and calculated among- and within-individual variance accounted for behavioural repeatability (R), providing a true assessment of individual personality traits

Level	Family	Species	Complexity type (high ~ low)	Personality/Behaviour ~ Habitat complexity	Repeatability	Reference
P	Cichlidae	*Amatitlania nigrofasciata*	plants vs. barren tanks; complex vs. open habitat choice tank	aggression ↑ dominant fish preferred complex habitat	R	Church and Grant 2019
B	Cichlidae	*Oreochromis niloticus*	gravel/glass balls/coloured pipes vs. barren tanks	anxiety ↓ exploration ↑ neophobia ↓	O	Tatemoto et al. 2021
P	Cottidae	*Cottus perifretum*	vegetation/rocks/other structures vs. open water/sand streams	aggression ↓	R	Kobler et al. 2011
B	Danionidae	*Danio rerio*	plants/rocks/sand vs. barren tanks	anxiety ↓ exploration ↑	O	Manuel et al. 2015
B	Danionidae	*Danio rerio*	colourful Lego bricks vs. barren tanks	exploration ↑ neophobia ↓	O	Gatto et al. 2022
B	Danionidae	*Danio rerio*	plants/gravel/shelter vs. one plant/barren tanks	activity ↑	O	Green and Swaney 2023
B	Danionidae	*Danio rerio*	colourful Lego bricks vs. barren tanks	activity ↑	M	Santacà et al. 2024
B	Danionidae	*Danio rerio*	plants/colourful marbles and pebbles/pipes/bubbles vs. barren tanks	sociability x	M	Flores-Prieto et al. 2024
B	Gadidae	*Gadus morhua*	plants/rocks/pebbles vs. barren tanks	boldness ↑ exploration ↑	O	Braithwaite and Salvanes 2005
B	Gadidae	*Gadus morhua*	plants/rocks/pebbles vs. barren tanks	activity ↓ aggression x boldness ↓ exploration ↑	M	Salvanes and Braithwaite 2005
B	Gasterosteidae	*Gasterosteus aculeatus*	plants/pebbles/plant pots vs. barren tanks	activity x boldness x neophobia x	M	Brydges and Braithwaite 2009
B	Gasterosteidae	*Gasterosteus aculeatus*	changing physical structure vs. barren tanks	activity x aggression ↓ exploration x	M	Álvarez-Quintero and Kim 2024
B	Gobiidae	*Gobiocypris rarus*	plants/gravel vs. barren tanks	anxiety x	O	Xu et al. 2022
B	Melanotaeniidae	*Melanotaenia duboulayi*	plants/gravel vs. barren tanks	activity ↑	O	Bibost et al. 2013
P	Poeciliidae	*Gambusia affinis*	plants vs. barren tanks	boldness ↓ exploration ↓ sociability ↑	R	Xu et al. 2021
B	Poeciliidae	*Gambusia affinis*	vegetation/sediments vs. urbanized/degraded streams	boldness ↓	O	Irwin et al. 2024
B	Poeciliidae	*Gambusia hubbsi*	mangrove/rock vs. mud	exploration ↑	O	Jenkins et al. 2021
B	Rivulidae	*Kryptolebias marmoratus*	plants/shelter vs. barren tanks	activity ↑ neophobia ↑	O	Berbel-Filho et al. 2020
B	Salmonidae	*Oncorhynchus mykiss*	submerged tree structures/overhead cover vs. barren hatchery	aggression ↑	M	Berejikian et al. 2000
B	Salmonidae	*Oncorhynchus mykiss*	submerged tree structures/overhead cover vs. barren hatchery	aggression ↑	M	Berejikian et al. 2001
B	Salmonidae	*Oncorhynchus mykiss*	plants/shelter/novel objects/pipes/gravel vs. barren tanks	anxiety x	O	Ahlbeck Bergendahl et al. 2016
B	Salmonidae	*Salmo salar*	enrichment structures vs. barren tanks	aggression ↓	M	Rosengren et al. 2017
P	Salmonidae	*Salmo salar*	boulders/gravel/cobble vs. gravel/cobble tanks	activity x aggression x	R	Church and Grant 2018
B	Salmonidae	*Salmo salar*	stones vs. barren tanks	activity x aggression x exploration ↑	M	Dunaevskaya et al. 2025
P	Salmonidae	*Salvelinus alpinus*	plants/rocks vs. barren tanks	boldness →	R	Dellinger et al. 2025
B	Salmonidae	*Salvelinus confluentus*	rock substrate vs. barren tanks	boldness ↑	O	Brignon et al. 2018
B	Sparidae	*Sparus aurata*	gravel vs. barren tanks	aggression ↓	M	Batzina and Karakatsouli 2012
B	Sparidae	*Sparus aurata*	ropes vs. barren tanks	exploration ↑	M	Arechavala‐Lopez et al. 2020
B	Scorpaenidae	*Sebastes schlegelii*	wild vs. hatchery fish	boldness ↑	O	Zhang et al. 2023
B	Xenocyprididae	*Opsariichthys bidens*	30% plants/90% pebbles/other structures vs. 10%plants/40% pebbles	aggression ↓	M	Lin et al. 2024

It is likely in many cases that both plasticity and habitat selection contribute to these sorts of results. In order to tease them apart, experimental approaches involving manipulation of the rearing environment are required. Fortunately, the captive environment experienced by hatchery-reared fishes provides the perfect opportunity to do just that, and many researchers have taken advantage of this context to examine the influence of enrichment on fish personality and behaviour. For example, Xu et al. (2021) reared newborn mosquitofish in different levels of habitat complexity and tested personality at sexual maturity. Fish reared in complex environments were shyer, less explorative, and more social than those reared in open environments. These results suggest that early experiences of habitat complexity can have long-lasting influences on personality traits. By comparison, Church and Grant (2018) raised juvenile Atlantic salmon in tanks of varying structural complexity and found that increasing habitat complexity did not favour certain personality types but it altered salmonid behaviour in terms of decreased activity and aggression levels in complex habitats.

### Impact of habitat complexity on fish behaviour within a species

Although our understanding of how habitat complexity influences fish personality traits is still limited, studies investigating inter-individual behavioural variation offer valuable proxies for underlying personality traits. For instance, structurally complex habitats reduced aggression in threespined sticklebacks (Álvarez-Quintero and Kim 2024), gilthead sea bream (Batzina and Karakatsouli 2012), Atlantic salmon (Rosengren et al. 2017), and the Chinese hooksnout carp (Lin et al. 2024) (see Table 3, see Fig. 2). On the other hand, juvenile steelhead trout reared in structurally enriched environments in captivity showed similar levels of aggression to wild fish, while fish reared in standard conditions were significantly less aggressive (Berejikian et al. 2000, 2001). Hatchery-reared black rockfish kept in barren flow-through tanks differed from wild fish caught along the rocky coast; they used shelter more often, were bolder, and took longer to respond to predatory threat (Zhang et al. 2023). Brignon et al. (2018) found that the rearing environment influenced a range of behavioural traits in bull trout, including boldness, with trout reared in complex environments being bolder than those reared in conventional (barren) conditions. Structural enrichment also enhanced exploratory behaviour in seabream and salmon (Arechavala-Lopez et al. 2020; Dunaevskaya et al. 2025), and decreased anxiety, while increasing exploration in tilapia and zebrafish (Manuel et al. 2015; Tatemoto et al. 2021). Nonetheless, studies also observed no impact of habitat complexity on behavioural variation in threespined sticklebacks, gudgeons, rainbow trouts, and zebrafish (Brydges and Braithwaite 2009; Ahlbeck Bergendahl et al. 2016; Xu et al. 2022; Flores-Prieto et al. 2024).

### Impact of habitat complexity on fish personality and behaviour; intergenerational and transgenerational effects

It is plausible that variation in behavioural and personality traits is also influenced by epigenetic mechanisms, including parental effects and other non-genetic inheritance pathways. Previous studies have demonstrated, for example, that boldness can be heritable, potentially mediated by epigenetic processes (Tulley and Huntingford 1987; Brown et al. 2007; Stein and Bell 2014; Berlinghieri et al. 2025). It seems likely that similar parental effects could also occur in the context of habitat complexity. To the best of our knowledge, only one study has examined the potential intergenerational impacts of habitat complexity on personality traits. In a recent study, Dellinger et al. (2025) used offspring of 5 wild-caught Arctic charr morphs, which experience variation in the spatiotemporal availability of structurally complex features within the lakes and show genetic divergence from a common ancestor, to investigate the impact of habitat complexity on personality traits. After hatching, juveniles were either raised in structurally enriched or barren tanks. Dellinger et al. (2025) discovered that boldness repeatability in juveniles depended more on the ecology of each morph rather than the habitat complexity treatment they were exposed to. Further, boldness tended to be more consistent in treatments mimicking natural complexity. The study confirms the dominant influence of genetic background over environmental factors on the development of boldness in this species, suggesting heritability of this personality trait (Dellinger et al. 2025). Apart from this study, no other papers have examined potential inter- or transgenerational impacts of habitat complexity on personality traits, although two come close. Berbel-Filho et al. (2020) examined the influence of habitat complexity on the parents and offspring of mangrove killifish (intergenerational). Parents reared in enriched habitats were more active and neophobic, and the offspring of these fish also matched their parents regardless of the environment they experienced. A study on zebrafish also found cross-generational effects of physical habitat enrichment on “the number of movement events” in F1 fish and also “total distance travelled” in F2 fish (transgenerational) (Green and Swaney 2023). It is worth noting, however, that no attempt was made to determine if these measures of behaviour were repeatable at the individual level, and neither of the two latter papers made any reference to animal personality. Further studies are clearly needed in this area.

## Linking habitat complexity with fish personality and cognition

Pavlov’s study on how distinctive and fixed behavioural phenotypes of different dogs would influence their associative learning abilities can be considered the first experiment linking animal personality traits with cognition (Gray and Eysenck 2017). Since then, several conceptual papers have explored the relationship between animal personality and cognition (Carere and Locurto 2011; Sih and Del Giudice 2012a, b; Griffin et al. 2015; Guillette et al. 2017), repeatedly proposing three central theories. The most popular prediction suggests a correlation between animal cognition and personality along the bold-aggressive-active-exploratory axis (Sih and Del Giudice 2012a, b). This theory proposes that more aggressive and bolder individuals are faster learners, which increases their chance to obtain a greater reward (e.g., more mates, food), but also carries a greater risk (e.g., predation, injury, false decisions). Other studies support the idea that fast explorers are also fast learners (i.e., associative learning), excelling in stable environments, whereas slow explorers tend to perform better in more variable environments (i.e., cognitive flexibility) (Verbeek et al. 1994; Guillette et al. 2009). A similar theory indicates that more explorative, bolder individuals are exposed more often to associations that can be made in their environment due to their personality, compared to less explorative, shyer individuals, influencing learning speed (Carere and Locurto 2011; Sih and Del Giudice 2012a, b; Griffin et al. 2015). An extensive meta-analysis by Dougherty and Guillette (2018) across a broad taxonomic range (i.e., insects, fish, reptiles, birds, and mammals) found indeed that animal personality and cognition are related, yet the direction of this relationship is highly variable.

A clear link between personality traits and cognitive styles has also been demonstrated in fish in laboratory settings. For instance, in threespined sticklebacks, mormyrid fish, and male guppies, bolder individuals outperformed shyer ones in an associative learning task (Dugatkin and Alfieri 2003; Kareklas et al. 2017; Bensky et al. 2017). However, studies also show that the benefits of certain personality traits depend on the cognitive task. Gibelli et al. (2019) showed in sailfin mollies that more anxious individuals performed worse at an associative learning task, but better in a spatial reversal task than less anxious fish. In another example, more active individuals in a western mosquitofish population were faster learners yet displayed a constrained memory (Liu et al. 2022). Strong evidence for covariation between cognition, personality/behavioural, life history, and physiological traits in teleost fishes has been provided by a series of studies by De Russi et al. (2024, 2025). For instance, in eels, distinct combinations of cognitive and personality traits were linked to alternative migratory strategies, emphasising an intrinsic connection to multidomain trait variance (De Russi et al. 2024). In guppies, a network analysis revealed sex-specific differences along a fast-slow continuum, with males expressing stronger covariation between personality and cognitive traits than females (De Russi et al. 2025). The authors highlight the importance of analysing multiple interacting traits when assessing fitness consequences in fishes, particularly in the context of improving conservation outcomes.

These studies suggest that, for instance, if habitat complexity alters personality traits, then, due to these covariations, cognition may also be expected to change. Yet, to the best of our knowledge, there is currently no research output that examined the effects of structural habitat complexity on fish personality traits (studies that account for behavioural repeatability) and the potential downstream consequences for cognitive abilities. Nonetheless, a handful of studies can provide an idea of how habitat complexity affects both personality traits and cognition in fish. Raising juvenile threespined sticklebacks in structurally complex tanks, for instance, improved motor inhibitory control abilities (general cognition), increased relative brain size, and decreased aggression in shoals (Álvarez-Quintero and Kim 2024). Arechavala‐Lopez et al. (2020) demonstrated that structural enrichment led to overall higher exploratory behaviour in gilthead seabream as well as enhanced spatial learning and memory capabilities (spatial cognition). These results align with the theories that fast explorers are also faster learners, potentially because they are exposed more quickly to associations that can be made in their environment. Studies on rainbow trout revealed that increased habitat complexity led to increased aggression in individuals, enabling individuals to reach significantly greater social dominance ranks, suggesting greater competitive ability of juveniles grown in the enriched tanks (Berejikian et al. 2000, 2001). Finally, in newly hatched zebrafish, structural habitat complexity promoted greater locomotor activity and individuals raised under these conditions outperformed individuals from barren tanks in a numerical discrimination task (numeracy) (Santacà et al. 2024).

## Future outlook

Empirical evidence suggests that species inhabiting structurally complex environments have evolved larger brains or expanded brain regions to meet the cognitive demands of these habitats, allowing them to process greater amounts of environmental information and to locate and exploit more diverse and heterogeneously distributed resources (Bennet and Harvey 1985; Petren and Case 1998; Safi and Dechmann 2005; Pamela Delarue et al. 2015; Steck and Snell-Rood 2018). In fishes, habitat complexity has played a key role in shaping brain structure over evolutionary timescales and through neural plasticity. Species occupying more structurally complex environments often possess relatively larger brains, telencephala, and cerebella (see Table 1, see Fig. 2). Comparable patterns have been reported in mammals (Harvey et al. 1980; Eisenberg and Wilson 1981; Budeau and Verts 1986; Safi and Dechmann 2005; Bertrand et al. 2017), birds (Bennet and Harvey 1985), and amphibians (Taylor et al. 1995; Liao et al. 2015). However, whether habitat complexity is a primary driver of relatively larger brains across taxa remains uncertain, as decades of research have found contradicting results (Clutton-Brock and Harvey 1980; Schuck-Paim et al. 2008; Powell et al. 2017; Heldstab et al. 2018). For instance, in lizards and snakes, habitat complexity appears unrelated to brain size (Powell and Leal 2014; De Meester et al. 2019).

While the positive correlation between relative brain size—or specific brain regions such as the telencephalon and cerebellum—and habitat complexity appears consistent across fish species, studies within species reveal more variable relationships. Nevertheless, several studies have shown that habitat complexity can influence brain development across life stages, with neural structures remaining plastic throughout an individual’s life. These trends suggest that, compared to other vertebrate groups, fishes may exhibit a more direct and flexible link between habitat complexity and neural investment. This, in turn, has important implications for management and conservation, as enhancing structural complexity (e.g., through habitat restoration or the addition of physical structures) could effectively support cognitive function and behavioural adaptability in fish populations.

Triki et al. (2024a), however, challenge the assumption that larger brains necessarily confer superior cognition. Their opinion piece argues that, for instance, the tenfold difference in relative brain size between endotherms (e.g., mammals, birds) and ectotherms (e.g., fishes, reptiles, amphibians) may primarily reflect quantitative cognition (more versus less) rather than qualitative (presence versus absence) as well as more advanced sensory–motor systems in endotherms rather than cognitive capacity. At the same time, emerging comparative evidence suggests that fishes may not possess general intelligence to the same extent as endotherms (Aellen et al. 2022; Guadagno and Triki 2024; Triki et al. 2024b). This may be attributed to the lifelong plasticity of the fish brain, which remains highly responsive to environmental conditions and is organised in a modular, task-specific manner, unlike the general-purpose machinery of mammalian brains. On the contrary, De Russi et al. (2025) reported covariation in cognitive abilities in migratory eels, with individuals, for instance, excelling in spatial learning also displaying enhanced problem-solving, high cognitive flexibility, and shorter time to solve different cognitive tasks, which could be an indicator for general intelligence in fish. Similar patterns have been observed in guppies (Prentice et al. 2022), where individual performance was positively correlated across different cognitive tasks assessing associative, reversal, and motor learning, suggesting among-individual variation with some individuals outperforming others in all three tasks. Hence, it remains unclear if fish possess general intelligence as some studies find strong correlations between cognitive functions at the individual level, while other studies have found no such evidence, potentially due to methodological differences or constraints. If many cognitive processes require less neural tissue than previously thought, and if fish possess more modular cognitive abilities that they can “boost” on demand while others remain dormant due to their high brain plasticity (Triki et al. 2024a), then ecological pressures, such as habitat simplification, may have serious consequences on neural investment and cognitive flexibility. Numerous studies report that increased habitat complexity enhances cognitive abilities in fish (see Table 2). In particular, habitat complexity is positively associated with spatial learning, memory, and various forms of general cognition. By contrast, research linking habitat complexity with social cognition and numerical discrimination remains limited, highlighting important avenues for future investigation. Given that aquatic habitats worldwide are becoming increasingly simplified, reductions in structural complexity could erode cognitive phenotypic plasticity within populations and species, ultimately constraining their ability to adapt to rapid environmental change. Moreover, the level of structural complexity fish encounter can vary spatiotemporally throughout their life cycle. Complexity may shift across space and time; sometimes predictably, as in species that migrate between structurally rich and poor environments, and sometimes unpredictably due to rapid environmental disturbances (e.g., bleaching events, storms). These spatiotemporal dynamics introduce an additional layer of environmental variability that may further shape cognitive abilities in fish.

Although we understand much of the direct effects of habitat complexity on fish cognition, far less is known about epigenetic effects whereby the habitat experienced by parents can affect their offspring or even grand offspring. Future research should therefore place greater emphasis on examining the transgenerational effects of habitat simplification on fish brain structure, personality and cognition. It may be that neural plasticity is so great in young fish that epigenetic effects of habitat complexity on brain morphology are masked. Moreover, our narrative review highlights that the ramifications of rearing fish in mundane environments extend beyond the contexts of aquaculture, fisheries, and conservation, into medical research (Volgin et al. 2018). Fish kept in impoverished environments may impact data validity and reliability, which could be detrimental, especially in medical research.

Habitat simplification may also have direct effects on an individual’s personality, e.g., changes in exploration, activity, neophobia, aggression, anxiety, and boldness (see Table 3), which in turn can shape cognitive styles, with profound implications for survival and fitness (Sih and Del Giudice 2012a, b; Rochais et al. 2022). Yet, studies investigating the intricate three-way interaction between habitat complexity, personality, and cognition in fishes are rather limited, highlighting an important direction for future investigations. Understanding these processes may enable us to better predict the outcomes of anthropogenic impacts such as climate change in physically complex ecosystems such as coral reefs.

Finally, this narrative review also highlights the importance of management, conservation, and restoration efforts to incorporate fish cognition into decisions about maintaining or reinstating habitat complexity. Considering cognition in these contexts could enhance ecosystem recovery. Additionally, future research should explore how habitat simplification and restoration affect fish cognition, personality, and welfare, and how cognitive changes in a single species may cascade through ecosystems by altering predator–prey dynamics, competition, or mating systems.

## Supplementary Information

Below is the link to the electronic supplementary material.

Supplementary file1

## Data Availability

No datasets were generated or analysed during the current study.
