# Supplementary material for: Simple life, simple minds? How habitat simplification in aquatic ecosystems shape fish cognition
Source: Anim Cogn. 2026 Feb 4;29(1):23. doi: 10.1007/s10071-025-02042-0 (PMC12876469; doi:10.1007/s10071-025-02042-0)
Supplement: Supplementary file 1 — Supplementary file1 [file 10071_2025_2042_MOESM1_ESM.docx]

**Simple life, simple minds? How habitat simplification in aquatic ecosystems shape fish cognition**

**Journal:**

Animal Cognition

**Author information:**

Annabell Klinke, Culum Brown

A. Klinke

Department of Biological Sciences, Macquarie University, Sydney 2109, Australia e-mail: [annabell.klinke@hdr.mq.edu.au](mailto:annabell.klinke@hdr.mq.edu.au) ORCiD: <https://orcid.org/0000-0002-1483-4837>

C. Brown

Department of Biological Sciences, Macquarie University, Sydney 2109, Australia e-mail: [culum.brown@mq.edu.au](mailto:culum.brown@mq.edu.au) ORCiD: <https://orcid.org/0000-0002-0210-1820>

**Supplementary Methods**

We utilized three databases to search for relevant studies: Google Scholar, Research Rabbit, and MultiSearch, which is provided by the Macquarie University Library. In Google Scholar, we designed three Boolean search strings to identify studies examining how habitat complexity affects brain structure, cognition, and personality in fish (refer to Table 1S). Research Rabbit is a visual literature mapping and discovery tool that assists researchers in exploring scholarly papers, authors, and connected research networks through interactive, citation-based graphs.

It is important to note that this is not a systematic review; therefore, we have not included every study published on these topics, but we do provide a well-balanced perspective. We established several criteria for including studies in our review. The primary criterion was that each study needed to investigate the relationship between physical habitat complexity and at least one measure of brain structure, cognition, or personality traits in fish.

Specifically, we included studies that focused on any aspect of brain structure, including morphological, structural, physiological, and molecular aspects. We also included studies that examined various areas of cognition, such as simple cognition, spatial cognition, social cognition, and numeracy. Additionally, since the concept of animal personality is relatively new, many earlier studies concentrated on behavioural variation rather than stable, individual traits. We only considered studies that measured individual behaviours multiple times and calculated both among- and within-individual variance to assess behavioural repeatability (R) as valid measures of animal personality traits (Dingemanse and Wright 2020). Nevertheless, we also included earlier studies that may not strictly qualify as animal personality research but still provide invaluable insights into the effects of habitat complexity on behavioural variability in fish. These studies are clearly identified as those in Table 3 and throughout the text. Moreover, we recognized anxiety as a personality trait, which is typically assessed through measures such as thigmotaxis, scototaxis, or freezing behavior in fish (Manuel et al. 2015; Gibelli et al. 2019). Finally, we did not specify definitions for each personality trait included in this review, as we deferred to the judgments of the original authors. We acknowledge that there is quite some discrepancy between papers in the assays used to assess personality in fishes, and some assays are used as indicators of multiple personality traits.

**Tab. 1S:** Boolean search strings used to identify literature on the relationship between habitat complexity, brain morphology, cognition, and personality in fishes.

| **Research theme** | **Search string (Google Scholar)** |
| --- | --- |
| **Habitat complexity and brain structure** | ("habitat complexity" OR "structural complexity" OR "physical enrichment" OR "environmental enrichment" OR "habitat enrichment") AND ("brain morphology" OR "brain structure" OR "neuroanatomy" OR "brain development") AND (“fish*”) |
| **Habitat complexity and cognition** | ("habitat complexity" OR "structural complexity" OR "physical enrichment" OR "environmental enrichment" OR "habitat enrichment") AND ("cognition" OR "cognitive performance" OR "cognitive ability" OR "simple learning" OR "associative learning" OR "spatial learning" OR "spatial memory" OR "working memory" OR "executive function" OR "cognitive flexibility" OR "reversal learning" OR "inhibitory control" OR "self-control" OR "numeracy" OR "social cognition" OR "problem solving") AND (“fish*”) |
| **Habitat complexity and personality** | ("habitat complexity" OR "structural complexity" OR "physical enrichment"  OR "environmental enrichment" OR "habitat enrichment") AND ("fish personality" OR "animal personality" OR "personality traits" OR "behavioural syndrome" OR "boldness" OR "shyness" OR "neophilia" OR "exploration" OR "sociability" OR "aggression" OR "activity" OR "risk-taking" OR "stress coping style" OR "coping style" OR “anxiety”) AND (“fish*”) |
